# Priming of Exogenous Salicylic Acid under Field Conditions Enhances Crop Yield through Resistance to *Magnaporthe oryzae* by Modulating Phytohormones and Antioxidant Enzymes

**DOI:** 10.3390/antiox13091055

**Published:** 2024-08-30

**Authors:** Wannaporn Thepbandit, Anake Srisuwan, Dusit Athinuwat

**Affiliations:** 1 Faculty of Science and Technology, Thammasat University, Pathumtani 12121, Thailand; wthep@tu.ac.th; 2 Faculty of Science and Technology, Nakhon Ratchasima Rajabhat University, Nakhon Ratchasima 30000, Thailand; 3 Center of Excellence in Agriculture Innovation Centre through Supply Chain and Value Chain, Thammasat University, Pathumtani 12121, Thailand

**Keywords:** indole-3-acetic acid, peroxidase, plant defense, plant’s immune response, rice blast, rice production, superoxide dismutase, systemic acquired resistance

## Abstract

This study explores the impact of exogenous salicylic acid (SA) alongside conventional treatment by farmers providing positive (Mancozeb 80 % WP) and negative (water) controls on rice plants (*Oryza sativa* L.), focusing on antioxidant enzyme activities, phytohormone levels, disease resistance, and yield components under greenhouse and field conditions. In greenhouse assays, SA application significantly enhanced the activities of peroxidase (POX), polyphenol oxidase (PPO), catalase (CAT), and superoxide dismutase (SOD) within 12–24 h post-inoculation (hpi) with *Magnaporthe oryzae*. Additionally, SA-treated plants showed higher levels of endogenous SA and indole-3-acetic acid (IAA) within 24 hpi compared to the controls. In terms of disease resistance, SA-treated plants exhibited a reduced severity of rice blast under greenhouse conditions, with a significant decrease in disease symptoms compared to negative control treatment. The field study was extended over three consecutive crop seasons during 2021–2023, further examining the efficacy of SA in regular agricultural practice settings. The SA treatment consistently led to a reduction in rice blast disease severity across all three seasons. Yield-related parameters such as plant height, the number of tillers and panicles per hill, grains per panicle, and 1000-grain weight all showed improvements under SA treatment compared to both positive and negative control treatments. Specifically, SA-treated plants yielded higher grain outputs in all three crop seasons, underscoring the potential of SA as a growth enhancer and as a protective agent against rice blast disease under both controlled and field conditions. These findings state the broad-spectrum benefits of SA application in rice cultivation, highlighting its role not only in bolstering plant defense mechanisms and growth under greenhouse conditions but also in enhancing yield and disease resistance in field settings across multiple crop cycles. This research presents valuable insights into the practical applications of SA in improving rice plant resilience and productivity, offering a promising approach for sustainable agriculture practices.

## 1. Introduction

Various factors, including unsuitable soil conditions, mineral deficiency, pests, and diseases, have a detrimental impact on rice (*Oryza sativa* L.) production, leading to reduced yields [1]. *Magnaporthe oryzae* causes blast disease, which is one of the most serious diseases affecting rice in Southeast Asia and Japan, resulting in average yield losses of 20% to 50% and even up to 100% in severe cases [2]. In Thailand, favorable climatic conditions facilitate the spread of rice blast, making it a particularly important threat to the country’s rice production systems. Consequently, it is crucial to prioritize measures that reduce the dissemination and intensity of rice blast to enhance yield and minimize losses in Thai rice production. Currently, farmers commonly use chemical fungicides, including Captan, Carbendazim, Thiram, or Tricyclazole, to control rice blast [3]. However, the use of these chemicals raises concerns about their potential negative effects on both humans and the environment [4]. Therefore, it is imperative to explore novel alternatives for effectively managing rice blast. Upon encountering potential pathogens or stressors, plants have the ability to activate their innate defense mechanisms in response [5]. These defense mechanisms protect the plant from damage and limit the spread of infections by employing a range of strategies. These include the production of antimicrobial compounds, strengthening of cell walls, and activation of signaling pathways that trigger systemic resistance [6]. Additionally, plants can increase the activity of antioxidant enzymes to mitigate oxidative stress caused by pathogen attack [7]. These elements not only shield the plant from intrusion but also provide structural support, enhancing its resilience [8]. By effectively coordinating these responses, plants enhance their ability to withstand and recover from pathogenic invasions, ensuring better growth and survival. The immunity of plants can be improved and their defense mechanisms enhanced through various approaches and elicitors [9]. Plant elicitors are substances or compounds that can induce or elicit defense responses in plants by mimicking signals associated with pathogen attack or environmental stress. Elicitors can be derived from various sources, including microbes, plants themselves, or synthetic chemicals, and they play a crucial role in enhancing plant immunity [10].

Exogenous salicylic acid (SA) is a strong elicitor that improves plant defense signaling, which causes more secondary metabolites to be made [11]. It achieves this by modulating the secondary metabolism biosynthetic pathway. Furthermore, exogenous SA can increase the activities of antioxidant enzymes, support making pathogenesis-related (PR) proteins, and cause antioxidant enzyme genes to be expressed in certain plant leaves [12]. There are numerous reports that SA priming is a crop protection method that uses the plant immune system. Research has shown that SA priming can improve resistance in maize (*Zea mays*) to fungal pathogens like *Fusarium*, which causes ear rot, leading to reduced mycotoxin contamination and better crop quality [13]. In wheat (*Triticum aestivum*), SA priming has been demonstrated to enhance resistance to rust diseases, caused by various species of *Puccinia* fungi, resulting in reduced disease severity and better grain yield [14].

Systemic acquired resistance (SAR) is a defense mechanism in plants that provides long-lasting, broad-spectrum resistance against a wide range of pathogens [15]. It is a systemic response that occurs in the entire plant, not only in the localized area of infection. Certain signaling molecules, such as SA, induce SAR upon exposure to pathogens or certain other stimuli. This enables the plant to defend against future pathogen attacks. Xi et al. [12] reported that the application of SA is an inducer of SAR in Pakchoi, which increases reactive oxygen species (ROS) levels, membrane lipid peroxidation, superoxide dismutase (SOD), ascorbic acid peroxidase (APX), catalase (CAT), and glutathione reductase (GR) under *Plasmodiophora brassicae* infection, which contributes to the reduction in club root diseases. These findings correlate with previous research that used exogenous SA as an inducer in rice, tomato, chickpea, grapevine, etc. [16,17,18]. In addition, SA and indole-3-acetic acid (IAA) play a significant role in plant physiology, including plant growth promotion and defense responses. While traditionally known as an important IAA involved in cell elongation, differentiation, and division, recent research has shed light on its role in SAR [19,20]. Both IAA and SA are involved in the regulation of ROS. ROS are signaling molecules that play a key role in plant defense. While SA can increase ROS production to enhance pathogen resistance, IAA can influence ROS levels to balance growth and defense, ensuring that energy is not overly diverted from growth processes to defense mechanisms [21,22]. Several studies have shown that exogenous SA can be used effectively in greenhouses to control rice disease, but its use in actual paddy fields has not been demonstrated. Additionally, priming rice with exogenous SA to induce resistance to blast disease has not been observed in local paddy fields. Therefore, the objective of this study was to examine the effects of exogenous SA priming on rice plants under field conditions, with a focus on modulating phytohormones and antioxidant enzymes that contribute to the defense responses of rice plants, improving resistance to *M. oryzae* and enhancing crop yield through these defense mechanisms.

## 2. Materials and Methods

### 2.1. Elicitor, Priming Conditions, and Cultivation

#### 2.1.1. Source of Elicitor and Priming Conditions

Exogenous SA from Krungthepchemi Co., Ltd., Bangkok, Thailand, was assessed at concentrations of 2 and 4 mM [23]. The SA was dissolved in distilled water and subsequently diluted to the desired concentrations, specifically 0.27624 g·L^−1^ for a 2 mM solution and 0.55248 g·L^−1^ for a 4 mM solution. For greenhouse conditions, the SA solution was prepared and applied to the rice plants by foliar spraying, with an application rate of 30 mL·pot^−1^. The treatments under investigation also involved the use of Mancozeb 80% WP with 30 g 20 L^−1^ of water, application rate of 30 mL·pot^−1^, which is conventionally treated by farmers to protect against natural infectious diseases, as the positive control [24], while the negative control consisted of water at a same application rate. For field trial conditions, a total volume of 20 L of the SA solution was prepared and applied to the rice plants by foliar spraying, with an application rate of 20 L·rai^−1^ (1 rai = 0.4 acres). The treatments under investigation also involved the use of Mancozeb 80% WP and water at a same application rate. The plants were treated with an exogenous SA solution and control solutions once a week for three weeks, starting on day 15 after planting. This treatment continued through the priming state, a phase where plants are exposed to a stimulus that enhances their ability to respond to future stresses more effectively.

#### 2.1.2. Greenhouse Conditions

The experiment was conducted in a completely randomized design (CRD) with 4 replications. The Thai jasmine rice cultivar ‘Khao Dowk Mali 105’ (KDML 105), which is susceptible to blast disease, was sterilized by soaking it in a 1% sodium hypochlorite solution for a duration time of 1 min and then rinsed with sterile water three times. Subsequently, the seeds were soaked in water for 24 h before planting. Ten seedlings were then transplanted into 10-inch plant pots filled with approximately 5 kg of sterile clay soil mixed with 5 g of nitrogen fertilizer. These pots were placed in a greenhouse under natural light conditions for 12 h at a temperature of 28 ± 4°C, with humidity maintained at 60–75%. The plants were treated with an exogenous SA solution and control solutions once a week for three weeks, using the concentration and application rate mentioned above, starting on day 15 after seed planting. A virulent strain of *M. oryzae* was supplied by Department of Agriculture, Ministry of Agriculture and Cooperatives, Thailand. *M. oryzae* was cultured on an oatmeal–rice agar medium at 25 ± 2 °C for 10 days. Conidia were collected by gently scraping the culture with a loop. The resulting suspensions were filtered through layers of cheesecloth to isolate pure conidia, and their density was adjusted to approximately 1 × 10^6^ conidia·mL^−1^ using a hemocytometer [25]. After sowing for 45 days, the rice plants were inoculated by spraying a suspension of *M. oryzae*. Antioxidant enzyme activity and phytohormone accumulation were observed at 12-h intervals for a total of 72 h post-inoculation (hpi).

#### 2.1.3. Field Trial Conditions

The experiment was conducted in a Randomized Complete Block Design (RCBD) with 4 treatments, 4 blocks, 3 replications per block. A field experiment was carried out in the local paddy field situated in Khok-Sung, Ubolratana District, Khon-Kaen Province, Thailand for a duration of 3 consecutive years during August-November 2021, 2022, and 2023. The soil in the area is classified as clay loam with a pH of 6.1, containing 0.92% soil organic matter and 0.01% nitrogen. Treatments and replications were separated by 1 m of buffer strips, measuring 40 cm in height. Each individual plot covered an area of 81 square meters (9 m by 9 m). The planting arrangement consisted of rows spaced 25 cm apart, with 32 rows and 32 seedlings per row in each plot [26,27]. The outer rows were assigned as boundary rows to minimize the possibility of sample cross-contamination. Seedlings were prepared for transplantation in a seedbed located in a small area, utilizing farm soil that had decomposed at a rate of 0.5 kg m^−2^ to a depth of 10 cm. The seeds were immersed in water for a duration time of 24 h and then planted in the seedbeds at a density of 50 g·m^−2^. After reaching 20 days of age, the seedlings were carefully removed from the ground and collected for transplantation, with a density of 3 stems per hill. The field was plowed in a zigzag pattern twice using a tractor 7 days before transplantation. A total of 500 kg of decomposition material per rai was applied 3 days prior to implantation. The fertilizers utilized adhered to the guidelines provided by the Land Department of Development (LDD), Ministry of Agriculture and Cooperatives, Thailand, which were based on the optimal levels of nitrogen, phosphorous, and potassium. A fertilizer formula with a nitrogen–phosphorus–potassium (NPK) ratio of 16–20–0 was used to fertilize the field during the tilling stage, starting from 10 days after transplantation. The application rate was 30 kg·rai^−1^. Additionally, a fertilizer formula with an NPK ratio of 0–0–60 was administered at a rate of 10 kg·rai^−1^. In addition, a 46–0–0 fertilizer formula was applied for 50 days following transplantation, namely during the panicle start stage, at a rate of 3 kg·rai^−1^. The weeds and golden apple snails were manually removed. Rice plants were foliar sprayed with exogenous SA and control solutions once a week for three weeks according to the concentration and application rate as described above, commencing 15 days after transplantation. The severity of rice blast disease (natural infection), growth characteristics, and yield production were evaluated by measuring one-square meter quadrants in each treatment with 12 replications to obtain representative data [27].

### 2.2. Antioxidant Enzyme Activity under Greenhouse Conditions

#### 2.2.1. Rice Leaf Sample Protein Extraction

Flag leaves from each treatment, 4 replications per treatment, and 3 plants per replication were harvested at 0 (pre-inoculation), 12, 24, 36, 48, 60, and 72 hpi to assess enzyme activity.

The leaf samples, with 1 g of fresh weight obtained by cutting and combining sections from different parts of the flag leaves to ensure a representative sample, were ground in liquid nitrogen (LN_2_) until they became powder. The powder was homogenized in 3 mL of phosphate buffer (0.1 M, pH 7.5). Then, the supernatant was transferred to a new tube containing 2 mL of trichloroacetic acid (TCA) solution at a concentration of 10% for protein precipitation. The precipitation protein was obtained by centrifugation at 12,000× *g* at 4 °C for 15 min. The protein content of each sample was examined using the standard Bradford protein assay [28]. The enzyme products were stored at 4 °C and used as a source for further experiments.

#### 2.2.2. Peroxidase (POX) Activity

The spectrophotometer was used to assess peroxidase activity, following the method provided by Hammerschmidt et al. (1982) [29]. The enzyme extract (0.1 mL) was mixed with a reaction mixture consisting of 1.5 mL of 0.05 M pyrogallol and 0.5 mL of 1% hydrogen peroxide (H_2_O_2_). The variation in absorbance was measured at 420 nm using a Bio-Tek microplate reader (Winooski, VT, USA) per min for a total of 3 min. A control mixture was made using nuclease-free water instead of the enzyme extract. The enzyme activity was quantified in units. The value was expressed as units·g^−1^·FW·min^−1^ [29]. The experiment on POX activity was conducted 3 times, with each experiment consisting of 4 replications.

#### 2.2.3. Polyphenol Oxidase (PPO)

The reaction was started by combining 1.5 mL of a 0.1 M solution of sodium phosphate (Na_3_PO_4_; pH 7.0) with 0.1 mL of an enzyme extract in a cuvette. Afterward, 0.2 mL of catechol (0.01 M) was introduced to start the reaction. The variation in absorbance was observed at a wavelength of 495 nm at intervals of 1 min for a duration of 3 min. The results were expressed as units·g^−1^·FW·min^−1^ [30]. The experiment on PPO activity was conducted 3 times, with each experiment consisting of 4 replications.

#### 2.2.4. Catalase (CAT) Activity

The CAT analysis reaction mixture consisted of 0.5 mL of 0.1 M phosphate buffer (pH 7.0), 0.1 mL of 1% H_2_O_2_, and 0.2 mL of enzyme extract. Following this, the mixture underwent incubation at 28 °C for 1 min. To establish a baseline, the absorbance of the mixture was zeroed at 230 nm using a Bio-Tek microplate reader (Winooski, VT, USA). Changes in absorbance were then monitored at 1 min intervals over a span of 3 min. The experiment on CAT activity was conducted 3 times, with each experiment consisting of 4 replications [31].

#### 2.2.5. Superoxide Dismutase (SOD)

The SOD activity was determined by assessing its capacity to stop the photochemical reduction of nitroblue tetrazolium (NBT). The combination of reactants comprised 50 mM phosphate buffer (pH 7.8), 20 µM riboflavin, 75 mM NBT, 13 mM methionine, and 0.1 mM ethylenediaminetetraacetic acid (EDTA). After illumination with two fluorescent light tubes for 10 min, absorbance was determined at 560 nm using a Bio-Tek microplate reader (Winooski, VT, USA) [32,33]. The experiment on SOD activity was conducted 3 times, with each experiment consisting of 4 replications.

### 2.3. Phytohormones Accumulation

Flag leaves from each treatment were harvested and immediately processed to prevent degradation of phytohormones at 0 (pre-inoculation), 12, 24, 36, 48, 60, and 72 hpi to assess endogenous SA and IAA accumulation in rice plant.

#### 2.3.1. Endogenous SA

The leaf samples were frozen in LN_2_ and then blended with 1 mL of a buffer solution consisting of 90% methanol, 9% glacial acetic acid, and 1% water. Following this, the samples underwent centrifugation at 14,000× *g*, 10 min at a temperature of 4 °C. After the process of centrifugation, 0.5 mL of the liquid above the sediment was moved to a 1.5 mL tube that had previously contained 0.5 mL of a solution with a concentration of 0.02 M of ferric ammonium sulphate. The mixture was then left to react for a duration of 5 min at a temperature of 30 °C. Afterwards, 200 µL of each sample was moved to a 96-well plate, and the amount of light absorbed was quantified at a wavelength of 530 nm using a Bio-Tek microplate reader located in Winooski, VT, USA. The amounts of endogenous SA were evaluated by comparing the absorbance results with standard benchmarks [34]. The experiment on endogenous SA activity was conducted 3 times, with each experiment consisting of 4 replications.

#### 2.3.2. Indole-3-Acetic Acid (IAA)

To measure the concentration of IAA using Salkowski’s reagent, prepare the reagent by mixing ferric chloride (FeCl_3_) with 10.8 M sulfuric acid (H_2_SO_4_). Then, mix 2 mL of supernatant with 3 mL of the prepared reagent, and incubate the mixture for 30 min in the dark. Next, measure at 550 nm using a spectrophotometer, and compare the OD value to a standard curve to determine the IAA concentration [35]. The experiment on IAA activity was conducted 3 times, with each experiment consisting of 4 replications.

### 2.4. Disease Index

The disease symptoms were evaluated on the rice leaves in greenhouse conditions for 5 times at 7, 14, 21, 28, and 35 days post-inoculation (dpi) using the IRRI standard for rice blast disease scoring, which outlines the following criteria (Table 1 [26]).

The effectiveness of blast disease management under field trial conditions was evaluated against natural *M. oryzae* infection following the IRRI standard (Table 1 [26]). Disease severity was assessed on 5 occasions starting from day 45 post-transplantation. The percentage of disease severity and reduction of both conditions were calculated using Equations (1) and (2).

(1) $$\frac{\Sigma (Rice\;blast\;rating\;\times \;Number\;of\;leaves\;of\;this\;rating)}{Total\;rice\;leaves\;\times \;maximum\;rating\;scale}\;\times \;100$$

(2) $$\frac{Control\;treatment\;-\;Treated\;treatment}{Control\;treatment}\;\times \;100$$

### 2.5. Yield Parameters

Plant parameters were monitored, including plant height, number of tillers and panicles per hill, number of seeds per panicle, and 1000-grain weight. Additionally, overall grain yield was assessed and reported as kg·rai^−1^ [36].

### 2.6. Statistical Analysis

The experimental data were subjected to one-way ANOVA using SPSS Software version 20. Mean differences were determined using Duncan’s multiple range test, with a significance level set at *p* ≤ 0.05.

## 3. Results

### 3.1. Antioxidant Enzyme Activity under Greenhouse Conditions

#### 3.1.1. Peroxidase (POX) Activity

**Figure 1:**
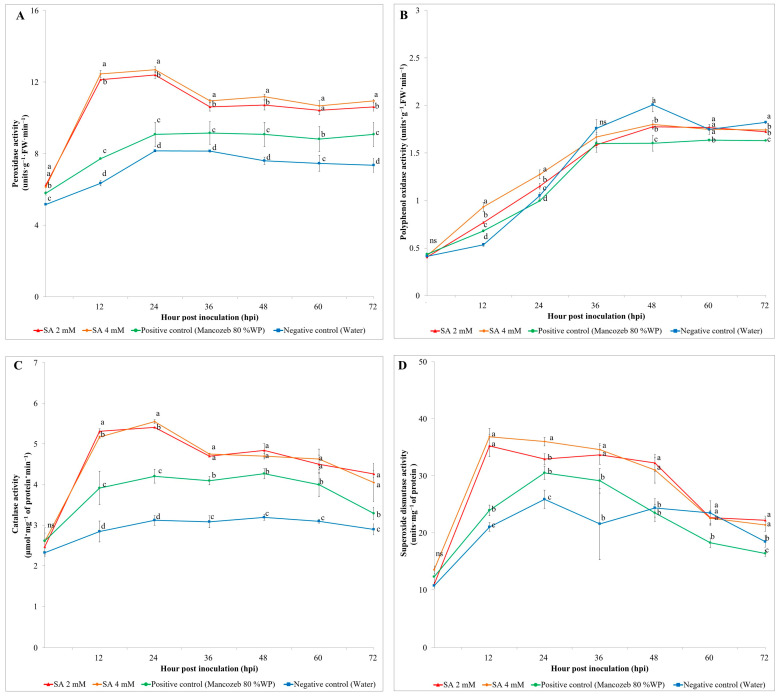
Effects of different treatments on antioxidant enzyme activity in plants at pre (0 h) and post inoculated with *Magnaporthe oryzae* over a 72-h period. (**A**) Peroxidase (POX) activity, (**B**) polyphenol oxidase (PPO), (**C**) catalase (CAT) activity, and (**D**) superoxide dismutase (SOD). Different letters (a, b, c, d) indicate significant differences via Duncan’s multiple range test at *p* ≤ 0.05. “ns” denotes non-significant differences.

The impact of two doses of exogenous SA, alongside positive (Mancozeb 80% WP) and negative control (water) samples, was assessed at both pre- and post-inoculation time points, on POX activity in rice plants. The application of SA spray resulted in a significant increase in POX activity at 12 hpi. Specifically, the highest POX activity was recorded in the 4 mM SA-treated sample, which reached approximately 12.70 units·g^−1^·FW·min^−1^, representing a three-fold increase compared to the initial level. Similarly, the 2 mM SA-treated sample showed a significant increase, peaking at 12.40 units·g^−1^·FW·min^−1^ at 12 hpi. These values were substantially higher than those observed in the positive control, which had a maximum POX activity of 7.72 units·g^−1^·FW·min^−1^, and the negative control, which peaked at 6.35 units·g^−1^·FW·min^−1^ (Figure 1A).

In contrast, the POX content in the negative and positive control samples reached their highest levels at 24 hpi, with values of 9.08 and 8.16 units·g^−1^·FW·min^−1^, respectively. After the initial peaks, POX activity in all treatments gradually declined but remained higher in the SA-treated samples compared to the controls throughout the observation period up to 72 hpi. The application of SA spray resulted in a significant increase in POX activity at 12 hpi.

#### 3.1.2. Polyphenol Oxidase (PPO)

The impact of two doses of exogenous SA on PPO activity is shown in Figure 1B. At 12 hpi, the application of SA began to significantly increase PPO activity. The 4 mM SA treatment showed an increase, and by 24 hpi, PPO activity reached approximately 1.28 units·g^−1^·FW·min^−1^. The 2 mM SA treatment followed a similar trend, with PPO activity rising to around 1.15 units·g^−1^·FW·min^−1^ by 24 hpi. In contrast, the positive control (Mancozeb 80% WP) showed a gradual increase in PPO activity, peaking at about 1.64 units·g^−1^·FW·min^−1^ at 48 hpi. The negative control (water) also exhibited a delayed response, with PPO activity reaching its highest level of approximately 2.01 units·g^−1^·FW·min^−1^ at 48 hpi. After 48 hpi, PPO activity in exogenous SA and the positive control treatments showed a gradual decline whereas negative control treatment showed maintained higher PPO activity.

#### 3.1.3. Catalase (CAT) Activity

Figure 1C illustrates the changes in CAT activity in response to exogenous SA application in rice plants. CAT activity in the 4 mM SA treatment started to increase significantly by 12 hpi, indicating a rapid response to SA application. By 24 hpi, CAT activity reached its peak at approximately 5.55 μmol·mg^−1^ of protein min^−1^. Similarly, the 2 mM SA treatment showed an initial increase in CAT activity starting at 12 hpi, with a peak activity of 5.41 μmol·mg^−1^ of protein min^−1^ at 24 hpi. In comparison, the positive control (Mancozeb 80% WP) exhibited a more gradual increase in CAT activity, reaching its maximum value of about 4.21 μmol·mg^−1^ of protein min^−1^ at 24 hpi. The negative control (water) also showed a delayed response, with CAT activity peaking at approximately 3.12 μmol·mg^−1^ of protein min^−1^ at 24 hpi. After 24 hpi, CAT activity in all treatments began to decline.

#### 3.1.4. Superoxide Dismutase (SOD)

The effects of exogenous SA on SOD activity in rice plants over time were explored. The SOD activity in the 4 mM SA treatment showed the highest increase, significantly increasing by 12 hpi, indicating an early response to SA application. By 12 hpi, the SOD activity peaked at approximately 36.84 units·mg^−1^ of protein. The 2 mM SA treatment also showed a significant increase in SOD activity starting at 12 hpi, reaching its peak at around 35.26 units·mg^−1^ of protein. In comparison, the positive control (Mancozeb 80% WP) exhibited a more gradual increase in SOD activity, peaking at about 30.54 units·mg^−1^ of protein at 24 hpi. The negative control (water) showed the lowest increase, with SOD activity peaking at around 25.89 units·mg^−1^ of protein at 24 hpi (Figure 1D).

### 3.2. Phytohormone Accumulation

#### 3.2.1. Endogenous SA

**Figure 2:**
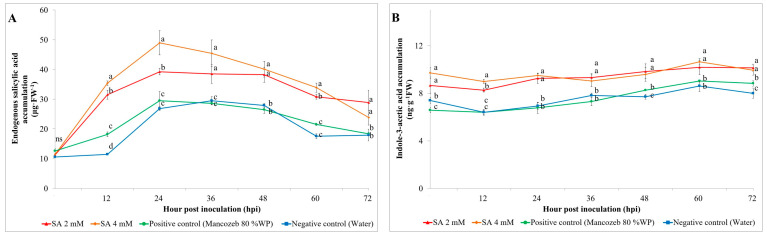
Effects of different treatments on endogenous salicylic acid and indole-3-acetic acid levels in plants at pre (0 h) and post inoculated with *Magnaporthe oryzae* over a 72-h period. (**A**) Quantification of endogenous SA content. (**B**) Quantification of IAA content. Different letters (a, b, c) indicate significant differences via Duncan’s multiple range test at *p* ≤ 0.05. “ns” denotes non-significant differences.

The influence of exogenous SA on the endogenous SA content in rice plants was investigated. The results showed that the exogenous SA-treated sample at dose of 4 mM showed the highest content of endogenous SA (48.98 μg·g^−1^ FW), followed by the exogenous SA-treated sample at dose of 2 mM (39.27 μg·g^−1^ FW) and positive control (Mancozab 80% WP)-treated sample (29.52 μg·g^−1^ FW). Meanwhile, the negative control (water)-treated sample showed the highest content of endogenous SA at 36 hpi with 29.54 μg·g^−1^ FW (Figure 2A).

The 4 mM SA treatment resulted in the highest accumulation of endogenous SA, peaking at 24 hpi with a value of approximately 48.98 μg·g^−1^ FW. This peak represents a significant increase from the initial levels measured at 0 hpi. Similarly, the 2 mM SA treatment showed a substantial increase in endogenous SA levels, peaking at around 39.27 μg·g^−1^ FW at 24 hpi. The positive control exhibited a more gradual increase in endogenous SA levels, reaching a maximum value of about 29.52 μg·g^−1^ FW at 24 hpi. In contrast, the negative control displayed the significantly lowest accumulation of endogenous SA, peaking at approximately 26.89 μg·g^−1^ FW at 24 hpi. After 24 hpi, endogenous SA levels began to decline in SA and positive control treatment, whereas water treatment was still increasing until 48 hpi. However, the SA-treated samples maintained higher levels compared to both negative and positive control treatments throughout the 72-h observation period.

#### 3.2.2. Indole-3-Acetic Acid (IAA)

The level of IAA was examined before and after inoculation with *M. oryzae*. The IAA level before inoculation for each treatment had a significantly different level. The results showed that the exogenous SA-treated samples at a dose of 4 mM showed the highest content of IAA with 9.71 ng·g^−1^ FW, followed by the exogenous SA-treated samples at a dose of 2 mM with 8.65 ng·g^−1^ FW, negative control (water)-treated samples with 7.39 ng·g^−1^ FW, and positive control (Mancozab 80% WP)-treated samples with 6.58 ng·g^1^ FW, respectively. After the inoculation, the 4 mM SA treatment showed a significant increase in IAA levels, starting at 24 hpi and continuing to increase, reaching approximately 9.93 ng·g^−1^ FW at 72 hpi. The 2 mM SA treatment also exhibited a notable increase peaking at around 10.15 ng·g^−1^ FW at 72 hpi. The positive control displayed a steady increase in IAA levels, reaching about 8.84 ng·g^−1^ FW at 72 hpi. The negative control showed the lowest accumulation of IAA, with a peak of 8.00 ng·g^−1^ FW at 72 hpi (Figure 2B).

### 3.3. The Efficacy of SA against Rice Blast under Greenhouse Conditions

The effectiveness of exogenous SA at two doses, 2 and 4 mM, was compared with a negative control and a positive control treated sample to inhibit rice blast disease under greenhouse experiments. Mancozeb 80% WP was utilized as the positive control, while water served as the negative control. The results revealed that the application of exogenous SA treatment before *M. oryzae* inoculation resulted in a decrease in disease severity when compared to both the positive and negative control treatments. This reduction in blast disease severity was evidenced by higher percentages of disease reduction. The presence of typical rice blast symptoms was observed, characterized by the appearance of elliptical lesions or white-gray patches that were encircled by dying brown margins. At 7 dpi, the positive control treatment revealed the lowest disease severity (8.22%), followed by treatments with 4 mM exogenous SA (10.70%), and 2 mM exogenous SA (12.29%); these values were significantly lower than those in the negative control treatment (16.70%). At 14 dpi, the disease severity increased in all the treatments. The positive control treatment revealed the lowest disease severity (10.31%) followed by treatments with 4 mM exogenous SA (15.68%), 2 mM exogenous SA (16.26%), and the negative control (31.25%). At 21 dpi, the positive control treatment revealed the lowest disease severity (15.94%), followed by treatments with 4 mM exogenous SA (19.44%), 2 mM exogenous SA (20.59%), and the negative control (40.25%). At 28 dpi, the positive control treatment revealed the lowest disease severity (18.11%) followed by treatments with 4 mM exogenous SA (20.74%), 2 mM exogenous SA (22.26%), and the negative control (61.13%). At 35 dpi, the positive control treatment revealed the lowest disease severity (19.63%) followed by treatments with 4 mM exogenous SA (24.43%) and 2 mM exogenous SA (25.11%), whereas the negative control revealed the highest disease severity, reaching 83.76% (Table 2).

### 3.4. The Efficacy of SA on Yield Components and Yield

#### 3.4.1. Plant Height

**Figure 3:**
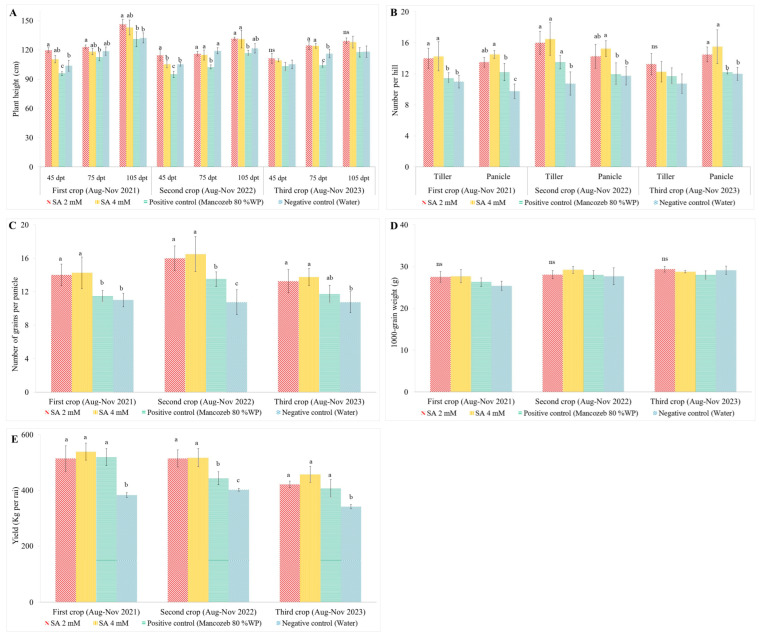
The effectiveness of exogenous salicylic acid elicitors on growth parameters and yield components of rice cv. KDML 105: (**A**) plant height; (**B**) number of tillers and panicles per hill; (**C**) number of grains per panicle; (**D**) 1000-grain weight; and (**E**) yield. Data presented as the mean ± SD (n = 12). Different letters (a, b, c) signify significant differences as determined by Duncan’s multiple range test at *p* ≤ 0.05. “ns” denotes non-significant differences.

Plant height significantly increased in samples treated with 2 and 4 mM exogenous SA across all three consecutive crops during August–November 2021, 2022, and 2023 for KDML 105 rice, compared to positive and negative controls. In the first crop, at 45 days post-transplantation (dpt), rice plants treated with 2 mM exogenous SA reached an average height of 119 cm, while those treated with 4 mM exogenous SA reached an average height of 111 cm. At 75 dpt, the 2 mM SA-treated plants showed an average height of 123 cm, and the 4 mM SA-treated plants reached 116 cm. By 105 dpt, the plants treated with 2 mM SA showed the highest height at 146 cm, followed by the 4 mM SA-treated plants at 143 cm. In the second crop, at 45 dpt, the 2 mM SA-treated plants had an average height of 114 cm, and the 4 mM SA-treated plants were 113.5 cm. At 75 dpt, the 2 mM SA-treated plants reached an average height of 116 cm, whereas the 4 mM SA-treated plants were 115 cm. At 105 dpt, the highest height recorded for 2 mM SA-treated plants was 131 cm, with 4 mM SA-treated plants close behind at 130 cm. In the third crop, at 45 dpt, the 2 mM SA-treated plants had an average height of 111 cm, while the 4 mM SA-treated plants reached 112 cm. At 75 dpt, the 2 mM SA-treated plants reached an average height of 124 cm, and the 4 mM SA-treated plants were 123 cm. At 105 dpt, the highest height recorded for 2 mM SA-treated plants was 129 cm, with 4 mM SA-treated plants measuring 128 cm. The data revealed that rice plants treated with 2 mM exogenous SA consistently showed the highest plant heights across all time points and crops, followed by plants treated with 4 mM exogenous SA. This indicates a clear positive effect of exogenous SA treatment on the growth of rice plants, with 2 mM concentration being the most effective (Figure 3A).

#### 3.4.2. Tillers and Panicles per Hill

In the first crop, rice plants treated with 4 mM exogenous SA exhibited the highest number of tillers per hill, averaging 14.25 tillers hill^−1^. This treatment outperformed both the positive and negative controls. Plants treated with 2 mM exogenous SA also showed a high number of tillers per hill, averaging 14 tillers hill^−1^. The tiller counts for SA treatments were higher than those of the control treatments, which showed significantly lower tiller numbers. In the second crop, the trend continued with the 4 mM exogenous SA-treated plants recording an average of 16.25 tillers hill^−1^, the highest among all treatments. The 2 mM SA-treated plants averaged 15 tillers hill^−1^, which was again higher compared to the controls. For the third crop, the highest number of tillers per hill was observed in plants treated with 2 mM exogenous SA, with an average of 13.25 tillers hill^−1^. This was not significantly different from the 4 mM SA-treated plants, which averaged 13 tillers hill^−1^. The negative control showed the lowest tiller counts across all crops. Regarding the number of panicles per hill, rice plants treated with 4 mM exogenous SA consistently exhibited the highest counts across all crops, with averages of 14.50, 15.25, and 15.50 panicles hill^−1^ in the first, second, and third crops, respectively. These values were not significantly different from those observed in the samples treated with 2 mM exogenous SA, which also showed high panicle counts. Conversely, the negative control treatment exhibited the fewest panicles per hill across all three crops. These results indicate that exogenous SA treatment, particularly at 4 mM, effectively enhances the number of tillers and panicles per hill in rice plants, contributing to improved plant vigor and potential yield benefits (Figure 3B).

#### 3.4.3. Grain per Panicle

The application of 4 mM exogenous SA resulted in a significant increase in the number of grains per panicle across all three crops studied, compared to the positive and negative control treatments. For the first crop, the plants treated with 4 mM SA exhibited an average of 14 grains panicle^−1^ as well as 2 mM SA-treated samples. Both treatments outperformed the negative control, which had significantly lower grains per panicle (11 grains panicle^−1^). In the second crop, the 4 mM SA treatment again resulted in the highest grain count per panicle, with an average of 16 grains panicle^−1^. The 2 mM SA-treated plants had an average of 15 grains panicle^−1^. These values were significantly higher than those observed in the positive and negative control treatments, which had lower grain counts (13 and 10 grains panicle^−1^, respectively). The trend continued in the third crop, where the 4 mM SA-treated plants recorded an average of 13 grains panicle^−1^. The 2 mM SA-treated plants had an average of 14 grains panicle^−1^, showing significantly higher values compared to the negative control treatment (11 grains panicle^−1^) (Figure 3C).

#### 3.4.4. Thousand-Grain Weight

The application of spray-applied exogenous SA resulted in the highest 1000-grain weight compared to both positive (Mancozeb) and negative (water) control samples across all three crops studied (Figure 3D). In the first crop, the 2 mM SA-treated plants exhibited an average 1000-grain weight of 27.50 g. The 4 mM SA-treated plants showed a slightly higher average weight of 27.69 g. These values were higher compared to the positive and negative control treatments (25.39 and 26.26 g, respectively). For the second crop, the 2 mM SA-treated plants had an average 1000-grain weight of 28.07 g, whereas the 4 mM SA-treated plants recorded a slightly higher average of 29.21 g, followed by positive and negative control treatments (28.07 and 27.64 g, respectively). In the third crop, the 2 mM SA-treated plants exhibited an average 1000-grain weight of 29.36 g, and the 4 mM SA-treated plants had an average weight of 28.78 g. Both SA treatments resulted in higher grain weights compared to the positive and negative control treatments (27.99 and 29.11 g, respectively). Although the difference among the treatments was not statistically significant, SA-treated rice plants showed higher weights compared to the controls.

#### 3.4.5. Yield

The application of 4 mM exogenous SA significantly increased grain yields across all three crops, yielding 539 kg rai^−1^ for the first crop, 517 kg rai^−1^ for the second crop, and 457 kg rai^−1^ for the third crop. This treatment demonstrated the highest yield compared to other treatments (Figure 3E). Following closely was the 2 mM exogenous SA treatment, which also resulted in substantial yield increases. The yields recorded for this treatment were 515 kg rai^−1^ for the first crop, 515 kg rai^−1^ for the second crop, and 422 kg rai^−1^ for the third crop. These figures represent significant improvements over the negative control, which yielded 383 kg rai^−1^ for the first crop, 402 kg rai^−1^ for the second crop, and 341 kg rai^−1^ for the third crop. The positive control, involving the application of Mancozeb 80% WP, produced yields of 519 kg rai^−1^ for the first crop, 443 kg rai^−1^ for the second crop, and 407 kg rai^−1^ for the third crop. While these yields were not significantly lower than those achieved with the exogenous SA treatments for the first and third crops, they were significantly lower for the second crop.

### 3.5. The Efficacy of Exogenous SA against Blast Disease under Field Conditions

**Figure 4:**
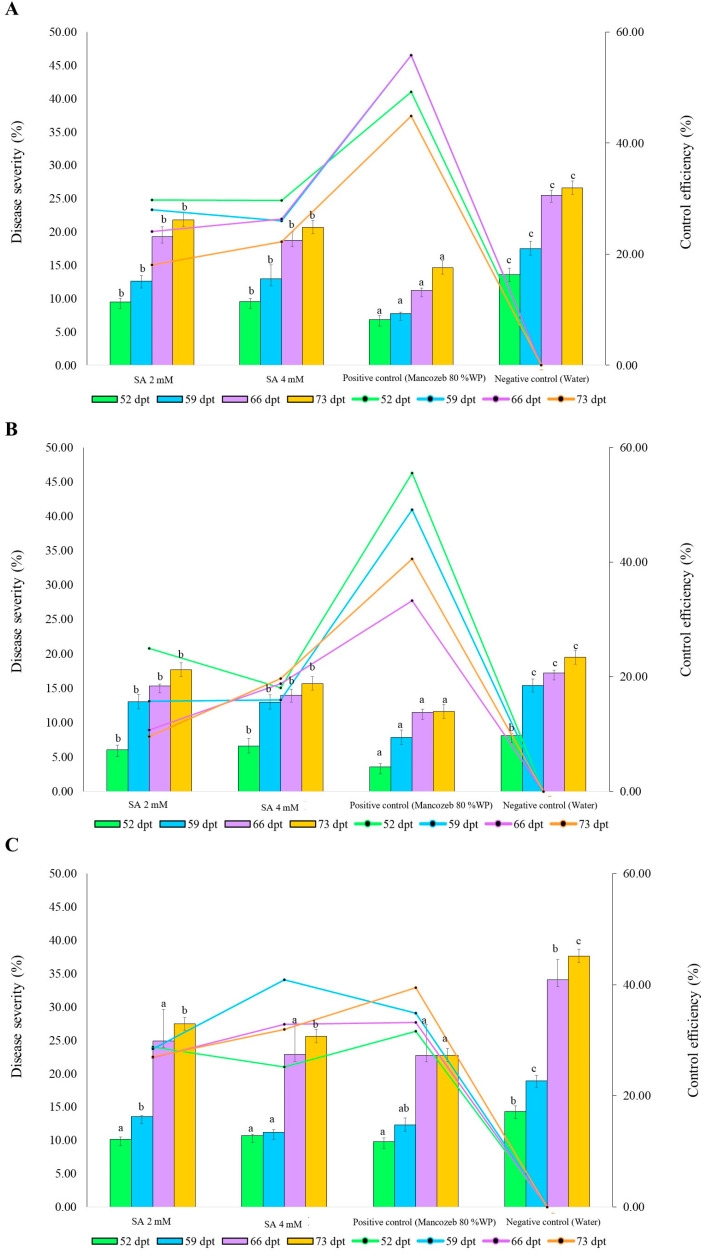
The efficacy of exogenous salicylic acid on the severity of rice blast disease on rice KDML 105 was evaluated during the following periods: (**A**) August–November 2021, (**B**) August–November 2022, and (**C**) August–November 2023. Data are presented as the mean ± SD (n = 12). Significant differences are denoted by different letters (a, b, c) according to Duncan’s multiple range test at *p* ≤ 0.05.

In the first crop, at 52 dpt, positive control (Mancozeb 80% WP) exhibited the highest reduction in blast disease severity. The average disease severity for Mancozeb was 6.89, which translates to a significant reduction of 49.00% compared to the negative control (water), which had an average severity of 13.57. The 2 mM SA treatment resulted in an average disease severity of 9.53, corresponding to a 29.81% reduction. The 4 mM SA treatment showed an average disease severity of 9.54, achieving a 29.70% reduction. At 59 dpt, the positive control treatment had an average disease severity of 7.73. This represents a 55.82% reduction compared to the negative control (17.50). The 2 mM SA treatment resulted in an average disease severity of 12.60, resulting in a 28.00% reduction. The 4 mM SA treatment showed an average disease severity of 12.95, achieving a 29.70% reduction. At 66 dpt, positive control treatment had an average disease severity of 11.26, showing a 55.82% reduction compared to the negative control treatment. The 2 mM SA treatment showed an average severity of 19.34, indicating a reduction of 24.09%. The 4 mM SA treatment showed an average disease severity of 18.76, leading to a 26.37% reduction. At 73 dpt, the positive control treatment had an average disease severity of 14.68, showing a 44.91% reduction in disease severity. The 2 mM SA treatment showed an average severity of 21.83, indicating a reduction of 18.07%. The 4 mM SA treatment had an average severity of 20.70, leading to a 22.25% reduction (Figure 4A). In the second crop, at 52 dpt, the positive control treatment exhibited the highest reduction in disease severity. The average disease severity for the positive control treatment was 3.59, which translates to a significant reduction of 55.55% compared to the negative control treatment, which had an average severity of 8.07. The 2 mM SA treatment resulted in an average disease severity of 6.06, corresponding to a 24.91% reduction. The 4 mM SA treatment showed an average disease severity of 6.62, achieving an 18.03% reduction. At 59 dpt, the positive control treatment had an average disease severity of 7.84. This represents a 55.55% reduction compared to the negative control (15.41). The 2 mM SA treatment resulted in an average disease severity of 12.99 resulting in a 15.68% reduction. The 4 mM SA treatment showed an average disease severity of 12.94, achieving a 15.99% reduction. At 66 dpt, the positive control treatment had an average disease severity of 11.46, showing a 33.28% reduction compared to the control. The 2 mM SA treatment showed an average severity of 15.33, indicating a reduction of 10.72%. The 4 mM SA treatment showed an average disease severity of 13.94, leading to an 18.81% reduction. At 73 dpt, the positive control treatment had an average disease severity of 11.61, showing a 40.53% reduction in disease severity. The 2 mM SA treatment showed an average severity of 17.66, indicating a reduction of 9.53%. The 4 mM SA treatment had an average severity of 15.68, leading to a 19.65% reduction (Figure 4B). In the third crop, at 52 dpt, the positive control treatment exhibited the highest reduction in disease severity. The average disease severity for the positive control treatment was 9.77, which translates to a significant reduction of 31.66% compared to the negative control, which had an average severity of 14.30. The 2 mM SA treatment resulted in an average disease severity of 10.18, corresponding to a 28.79% reduction. The 4 mM SA treatment showed an average disease severity of 10.69, achieving a 25.23% reduction. At 59 dpt, the positive control treatment had an average disease severity of 12.31. This represents a 34.91% reduction compared to the negative control (18.91). The 2 mM SA treatment resulted in an average disease severity of 13.53, resulting in a 28.47% reduction. The 4 mM SA treatment showed an average disease severity of 11.18, achieving a 40.89% reduction. At 66 dpt, the positive control treatment had an average disease severity of 22.76, showing a 33.21% reduction compared to the control. The 2 mM SA treatment showed an average severity of 24.93, indicating a reduction of 26.85%. The 4 mM SA treatment showed an average disease severity of 22.86, leading to a 32.91% reduction. At 73 dpt, the positive control treatment had an average disease severity of 22.78, showing a 39.51% reduction in disease severity. The 2 mM SA treatment showed an average severity of 27.47, indicating a reduction of 27.05%. The 4 mM SA treatment had an average severity of 25.62, leading to a 31.97% reduction (Figure 4C).

## 4. Discussion

The role of antioxidant enzyme activity in plant defense in plant disease control, where increased superoxide dismutase (SOD) activity contributes to cellular protection and defense regulation. Subsequently, the actions of SOD, catalase (CAT), and peroxidase(POX) engage in the detoxification of hydrogen peroxide, a byproduct of SOD activity. Catalase decomposes H_2_O_2_ into water and oxygen, while POX utilizes H_2_O_2_ in the oxidation of various substrates, aiding in pathogen resistance and the fortification of cell walls [37]. This dual approach to H_2_O_2_ detoxification is evident in the defense responses of rice to the blast fungus *M. oryzae*, where increased activities of CAT and POX are instrumental in maintaining cellular balance and enhancing structural defenses [38,39,40]. Parallelly, polyphenol oxidase (PPO) activates to catalyze the transformation of phenolic compounds into quinones (oxidizing agents) which are detrimental to many pathogens [41,42]. This enzymatic action not only reinforces the plant‘s physical barriers but also creates a hostile environment for the invading pathogens, where PPO-mediated browning serves as an effective defense strategy following mechanical damage or a pathogen attack [43]. In our results showed that the initial protection in PPO activity in SA-treated plants at 12–24 hpi represents a quick and robust defense response. In contrast, the higher PPO activity in water-treated plants at 36 hpi indicates a delay as the plant tries to manage the infection over a longer period. Moreover, it is noteworthy that PPO activity in water-treated plants showed the highest PPO accumulation over SA-treated plants throughout the 72 hpi observation period. PPO acts on phenolic compounds, producing quinones, which are toxic to pathogens. However, excessive production of quinone can also potentially reduce plant growth if not properly managed. In SA-treated plants, a rapid but short-lived spike in PPO activity was sufficient to halt the infection early, reducing the need of energy for sustained high levels of the enzyme. Thus, after the initial defense response, PPO activity is downregulated to conserve resources once the infection is mitigated.

The localized defense responses and detoxification processes involving ROS and antioxidative enzymes lead to the production of signaling molecules, such as SA, which facilitate the systemic transduction of defense signals throughout the plant [44,45]. This statement aligns with our findings, particularly highlighting the enhancement in plant immunological parameters, notably the elevation in endogenous SA levels (*p* ≤ 0.05). This systemic acquired resistance (SAR) is a hallmark of plant immunity, where defense signals activated by local pathogen attacks or ROS generation are propagated to distant tissues, initiating widespread defense responses [45,46]. In the aftermath of the immediate defense phase, plants embark on activating long-term defense strategies that encompass the synthesis of antimicrobial compounds, cell wall reinforcement, and the upregulation of defense-related genes [47,48]. The continuous engagement of antioxidative enzymes like POX and CAT in this phase is critical in ensuring cellular homeostasis and aiding in the recovery and healing of affected plant tissues [49]. Similarly, our results showed that the SA-treated samples maintained higher levels of POX and CAT activity compared to the control samples throughout the 72 hpi observation period. This suggests that exogenous SA application triggers a more robust and sustained increase in CAT activity, enhancing the plant’s ability to decompose hydrogen peroxide and mitigate oxidative stress. In addition, this study reveals a significant accumulation of indole-3-acetic acid (IAA). This indicates a complex interplay between SA and other hormonal pathways, influencing plant growth and stress responses. The increase in endogenous SA content suggests the amplification of the SA-mediated signaling pathway, while the modulation of IAA levels may affect various aspects of growth and development, including cell elongation and division. Unlike the trends observed for endogenous SA, IAA levels continued to increase throughout the 72 hpi observation period in all treatments. The highest and sustained increase in IAA levels in SA-treated samples suggests that exogenous SA application positively influences the accumulation of IAA, an important plant growth hormone, contributing to enhanced growth and stress responses. IAA influences cell wall composition and structure, which can affect the plant’s physical barriers to pathogen invasion. A stronger or more resilient cell wall can prevent or slow down the penetration and spread of pathogens [50,51]. Pathogen invasion is a form of biotic stress, and plants often increase IAA levels as part of their stress response to activate various defense mechanisms [52]. Increased IAA can promote root length and development, improving nutrient and water uptake, which strengthens the plant’s overall health and resistance to pathogens. Higher IAA levels can help in the fine-tuning of defense pathways, ensuring a robust and effective response to pathogen attacks by modulating the activities of other hormones like SA and JA [53]. This increase in IAA may be part of a feedback mechanism where initial pathogen detection leads to auxin accumulation, which then enhances the defense responses and prepares the plant for potential subsequent attacks. Additionally, some studies suggest that increased IAA levels can have direct antimicrobial effects, helping to suppress the growth and spread of pathogens within the plant tissues [54,55,56]. Our greenhouse experiments indicate that the application of exogenous SA at doses of 2 and 4 mM effectively controlled blast disease. The use of exogenous SA resulted in a significant reduction in blast disease severity, exhibiting a control effect of 47–51% compared to the negative samples. This observation is consistent with the findings of Shasmita et al. (2019), who reported that the application of 2 mM of SA positively influenced the reduction in bacterial blight disease by up to 69%. This reduction was attributed to the accumulation of defense-related enzymes [57]. 

**Figure 5:**
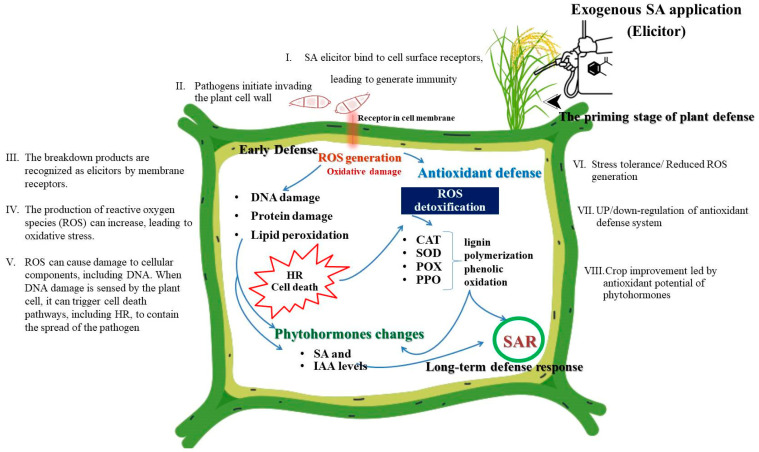
A proposed schematic model of antioxidative enzymes and hormonal responses in plant defense against *Magnaporthe oryzae* after being treated with exogenous salicylic acid.

The extension of these findings to field conditions across three consecutive crop seasons further validates the efficacy of exogenous SA in a regular agricultural environment. This study demonstrated the exogenous SA treatment’s consistent reductions in blast disease severity in all three crops. The study provides the beneficial effects of exogenous SA application on rice plants, as shown on a schematic model in Figure 5. The process begins with the application of exogenous SA, which binds to cell surface receptors. This binding initiates the priming stage of plant defense, preparing the plant for a more robust response upon *M. oryzae* attack. As *M. oryzae* invade, they break through the plant cell wall, marking the recognition stage of the defense process. The breakdown products from this invasion are recognized as elicitors by membrane receptors, which activate signaling pathways to prepare the plant for defense. Following elicitor recognition, the production of ROS increases, leading to oxidative stress. ROS are a crucial part of the plant’s early defense mechanism, helping to combat the pathogen. ROS can inflict significant damage on cellular components, including DNA, proteins, and lipids, resulting in oxidative stress. In response to oxidative damage, the plant’s early defense mechanisms are activated, leading to DNA damage, protein damage, and lipid peroxidation. These effects can trigger the hypersensitive response (HR) and cell death, which help contain the pathogen by sacrificing some plant cells to create a barrier. Changes in phytohormone levels, particularly in SA and IAA, help modulate the defense responses and prepare the plant for longer-term strategies. To counteract the oxidative damage caused by ROS, the plant activates its antioxidant defenses. Key enzymes involved in this process include CAT, which breaks down hydrogen peroxide into water and oxygen; SOD, which converts superoxide radicals into hydrogen peroxide; POX, which reduces hydrogen peroxide using various electron donors; and PPO, which oxidizes phenolic compounds to quinones, contributing to lignification and strengthening of cell walls. These act as secondary messengers in the SAR signaling pathway. SAR involves systemic signaling leading to long-term defense responses throughout the plant. This stage includes changes in gene expression and the production of various defense compounds, enhancing the plant’s overall resistance against future attacks. Finally, the upregulation or downregulation of antioxidant defense systems can lead to improved stress tolerance and reduced ROS generation. These changes ultimately enhance crop resilience and productivity. Furthermore, the results showed a clear decrease in disease severity, coupled with the observed improvements in yield components such as plant height, tillers, panicles, grain per panicle, and overall yield. These observed improvements were expected since a decrease in disease severity often leads to improvements in yield parameters. Healthier plants with lower disease levels can maintain a larger functional leaf area, enabling more efficient photosynthesis, which directly supports growth and development, enhancing yield [58]. When plants are less burdened by disease, they can allocate more energy and resources to growth and reproduction instead of defense and repair, leading to increased yield components [59]. Furthermore, healthier plants are generally more vigorous, allowing them to better withstand adverse environmental conditions. This increased resilience translates into improved growth and a potential increase in yield. In addition, lower disease levels mean that plants experience less stress, which can inhibit growth and development, thus contributing to an overall better plant health and improved yield parameters [60]. Several researchers reported that many plant diseases have a direct impact on yield components by damaging or reducing the size and quality of seeds, fruits, or grains [61]. By controlling disease severity, the quality and quantity of yield are preserved, resulting in significant improvements in yield parameters, as stated in this study.

## 5. Conclusions

This study elucidates the beneficial effects of exogenous SA application on rice plants, emphasizing its role in enhancing antioxidative defenses, modulating phytohormone levels, reducing disease severity, and improving yield components. These findings contribute valuable insights into the mechanistic understanding of SA-mediated responses in plants and highlight the potential of SA as an integral component of integrated plant disease management strategies in rice production. Further research is required to explore the underlying molecular mechanisms and to optimize SA application strategies for broader agricultural applications.

## Figures and Tables

**Table 1:** Rice blast disease severity rating scale.

Rice Blast Disease Scale	Infection of Plant
0	No symptoms are evident.
1	Small brown spots, resembling pinheads in size.
2	Large brown spots.
3	An oval necrotic gray speckle, measuring 1–2 mm in diameter, with a brown edge surrounding it.
4	There is an elliptical necrotic gray spot measuring 1–2 cm in length, indicative of symptoms affecting less than 2% of rice leaf area.
5	Symptoms are observed on less than 10% of rice leaf surface.
6	Symptoms are observed on 10% to 25% of rice leaf surface.
7	Symptoms are observed on 26% to 50% of rice leaf surface.
8	Symptoms are observed on 51% to 75% of rice leaf surface.
9	The entire leaf has died.

**Table 2:** The efficacy of salicylic acid (SA) on reducing the severity of blast disease in rice cv. KDML 105 caused by *Magnaporthe oryzae*.

Treatments	Disease Severity (%)					Control Efficiency (%)				
	7 dpi	14 dpi	21 dpi	28 dpi	35 dpi	7 dpi	14 dpi	21 dpi	28 dpi	35 dpi
2 mM SA	12.29 ± 0.27 ^a^	16.26 ± 0.28 ^b^	20.59 ± 1.72 ^b^	22.26 ± 1.88 ^b^	25.11 ± 1.50 ^b^	26.36	47.95	48.84	63.59	70.02
4 mM SA	10.70 ± 0.36 ^a^	15.68 ± 0.52 ^b^	19.44 ± 0.94 ^b^	20.74 ± 0.81 ^b^	24.43 ± 0.54 ^b^	35.91	49.83	51.7	66.07	70.83
Mancozeb 80% WP	8.22 ± 0.06 ^a^	10.31 ± 0.10 ^a^	15.94 ± 0.42 ^a^	18.11 ± 0.54 ^a^	19.63 ± 0.12 ^a^	50.76	67	60.39	70.37	76.56
Control (water)	16.7 ± 0.80 ^b^	31.25 ± 1.16 ^c^	40.25 ± 2.55 ^c^	61.13 ± 1.50 ^c^	83.76 ± 1.48 ^c^					

Data are mean ± SD (n = 4). Different letters (a, b, c) indicate significant differences via Duncan’s multiple range test at *p* ≤0.05.

## Data Availability

The original contributions presented in the study are included in the article, further inquiries can be directed to the corresponding author.

## References

[1] Reynolds T.W., Waddington S.R., Anderson C.L., Chew A., True Z., Cullen A. (2015). Environmental impacts and constraints associated with the production of major food crops in Sub-Saharan Africa and South Asia. Food Secur..

[2] Asibi A.E., Chai Q., Coulter J.A. (2019). Rice blast: A disease with implications for global food security. Agronomy.

[3] Moktan R., Aryal A., Karki S., Devkota A., Acharya B., Joshi D., Aryal K. (2021). Evaluation of different chemical fungicides against rice blast in field conditions. J. Agric. Nat. Resour..

[4] Ahmad M.F., Ahmad F.A., Alsayegh A.A., Zeyaullah M., AlShahrani A.M., Muzammil K., Saati A.A., Wahab S., Elbendary E.Y., Kambal N. (2024). Pesticides impacts on human health and the environment with their mechanisms of action and possible countermeasures. Heliyon.

[5] Li P., Lu Y.J., Chen H., Day B. (2020). The lifecycle of the plant immune system. CRC Crit. Rev. Plant Sci..

[6] Ding L.-N., Li Y.-T., Wu Y.-Z., Li T., Geng R., Cao J., Zhang W., Tan X.-L. (2022). Plant disease resistance-related signaling pathways: Recent progress and future prospects. Int. J. Mol. Sci..

[7] Kasote D.M., Katyare S.S., Hegde M.V., Bae H. (2015). Significance of antioxidant potential of plants and its relevance to therapeutic applications. Int. J. Biol. Sci..

[8] Freeman B., Beattie G. (2008). An overview of plant defenses against pathogens and herbivores. Plant Health Instr..

[9] Sirisha V.L., Mitra S., Suprasanna P., Fink G. (2024). Chapter 2—Plant immune system: Mechanisms and resilience. Stress: Immunology and Inflammation.

[10] Thakur M., Sohal B.S. (2013). Role of elicitors in inducing resistance in plants against pathogen infection: A review. ISRN Biochem..

[11] Jeyasri R., Muthuramalingam P., Karthick K., Shin H., Choi S.H., Ramesh M. (2023). Methyl jasmonate and salicylic acid as powerful elicitors for enhancing the production of secondary metabolites in medicinal plants: An updated review. Plant Cell Tissue Organ. Cult..

[12] Xi D., Li X., Gao L., Zhang Z., Zhu Y., Zhu H. (2021). Application of exogenous salicylic acid reduces disease severity of *Plasmodiophora brassicae* in pakchoi (*Brassica campestris* ssp. *chinensis* Makino). PLoS ONE.

[13] Li Z., Xu J., Gao Y., Wang C., Guo G., Luo Y., Huang Y., Hu W., Sheteiwy M.S., Guan Y. (2017). The synergistic priming effect of exogenous salicylic acid and H_2_O_2_ on chilling tolerance enhancement during maize (*Zea mays* L.) seed germination. Front. Plant Sci..

[14] Mapuranga J., Chang J., Zhao J., Liang M., Li R., Wu Y., Zhang N., Zhang L., Yang W. (2023). The underexplored mechanisms of wheat resistance to leaf rust. Plants.

[15] Métraux J.P., Maloy S., Hughes K. (2013). Systemic Acquired Resistance. Brenner’s Encyclopedia of Genetics.

[16] War A.R., Paulraj M.G., War M.Y., Ignacimuthu S. (2011). Role of salicylic acid in induction of plant defense system in chickpea (*Cicer arietinum* L.). Plant Signal Behav..

[17] Thepbandit W., Papathoti N.K., Daddam J.R., Hoang N.H., Le Thanh T., Saengchan C., Buensanteai K. (2023). In vitro and in silico studies of salicylic acid on systemic induced resistance against bacterial leaf blight disease and enhancement of crop yield. J. Integr. Agric..

[18] Singh S.K., Singh A., Dwivedi P. (2017). Modulating effect of salicylic acid in tomato plants in response to waterlogging stress. Int. J. Agric. Environ. Biotechnol..

[19] Ali A., Shah L., Ur Rehman S., Riaz M., Yahya M., Yunjian X., Liu F., Si W., Jiang H., Cheng B. (2018). Plant defense mechanism and current understanding of salicylic acid and NPRs in activating SAR. Physiol. Mol. Plant Pathol..

[20] Remans R., Spaepen S., Vanderleyden J. (2006). Auxin signaling in plant defense. Science.

[21] Huot B., Yao J., Montgomery B.L., He S.Y. (2014). Growth–defense tradeoffs in plants: A balancing act to optimize fitness. Mol. Plant.

[22] Zeier J. (2013). New insights into the regulation of plant immunity by amino acid metabolic pathways. Plant Cell Environ..

[23] Koo Y.M., Heo A.Y., Choi H.W. (2020). Salicylic acid as a safe plant protector and growth regulator. Plant Pathol. J..

[24] Kongcharoen N., Kaewsalong N., Dethoup T. (2020). Efficacy of fungicides in controlling rice blast and dirty panicle diseases in Thailand. Sci. Rep..

[25] Cai M., Miao J., Chen F., Li B., Liu X. (2021). Survival cost and diverse molecular mechanisms of *Magnaporthe oryzae* isolate resistance to epoxiconazole. Plant Dis..

[26] International Rice Research Institute (IRRI) (1996). Standard Evaluation System for Rice.

[27] IRRI (2013). Standardization Evaluation System for Rice.

[28] Bradford M.M. (1976). A rapid and sensitive method for the quantitation of microgram quantities of protein utilizing the principle of protein-dye binding. Anal. Biochem..

[29] Hammerschmidt R., Nuckles E., Kuć J. (1982). Association of enhanced peroxidase activity with induced systemic resistance of cucumber to *Colletotrichum lagenarium*. Physiol. Plant Pathol..

[30] Ainsworth E.A., Gillespie K.M. (2007). Estimation of total phenolic content and other oxidation substrates in plant tissues using Folin–Ciocalteu reagent. Nat. Protoc..

[31] Charoenphun N., Pham N.H., Rattanawut J., Venkatachalam K. (2024). Exogenous application of melatonin on the preservation of physicochemical and enzymatic qualities of pepper fruit from chilling injury. Horticulturae.

[32] Beauchamp C., Fridovich I. (1971). Superoxide dismutase: Improved assays and an assay applicable to acrylamide gels. Anal. Biochem..

[33] Gogliettino M., Arciello S., Cillo F., Carluccio A.V., Palmieri G., Apone F., Ambrosio R.L., Anastasio A., Gratino L., Carola A. (2022). Recombinant expression of archaeal superoxide dismutases in plant cell cultures: A sustainable solution with potential application in the food industry. Antioxidants.

[34] Raskin I., Turner I.M., Melander W.R. (1989). Regulation of heat production in the inflorescences of an Arum lily by endogenous salicylic acid. Proc. Natl. Acad. Sci. USA.

[35] Gang S., Sharma S., Saraf M., Buck M., Schumacher J. (2019). Analysis of indole-3-acetic acid (IAA) production in klebsiellaby LC-MS/MS and the salkowski method. Bio Protoc..

[36] Regalado M., Ramos P. (2018). Field testing of a rice crop postharvest management protocol for reduced postproduction losses and improved product quality. Rice-Based Biosyst. J..

[37] Passardi F., Cosio C., Penel C., Dunand C. (2005). Peroxidases have more functions than a Swiss army knife. Plant Cell Rep..

[38] Thepbandit W., Srisuwan A., Siriwong S., Nawong S., Athinuwat D. (2023). *Bacillus vallismortis* TU-Orga21 blocks rice blast through both direct effect and stimulation of plant defense. Front. Plant Sci..

[39] Gupta D.R., Khanom S., Rohman M.M., Hasanuzzaman M., Surovy M.Z., Mahmud N.U., Islam M.R., Shawon A.R., Rahman M., Abd-Elsalam K.A. (2021). Hydrogen peroxide detoxifying enzymes show different activity patterns in host and non-host plant interactions with *Magnaporthe oryzae Triticum* pathotype. Physiol. Mol. Biol. Plants.

[40] Liu X., Zhang Z. (2022). A double-edged sword: Reactive oxygen species (ROS) during the rice blast fungus and host interaction. FEBS J..

[41] Taranto F., Pasqualone A., Mangini G., Tripodi P., Miazzi M.M., Pavan S., Montemurro C. (2017). Polyphenol oxidases in crops: Biochemical, physiological and genetic aspects. Int. J. Mol. Sci..

[42] Vanitha S.C., Niranjana S.R., Umesha S. (2009). Role of phenylalanine ammonia lyase and polyphenol oxidase in host resistance to bacterial wilt of tomato. J. Phytopathol..

[43] Sullivan M.L. (2014). Beyond brown: Polyphenol oxidases as enzymes of plant specialized metabolism. Front. Plant Sci..

[44] Kapoor D., Singh S., Kumar V., Romero R., Prasad R., Singh J. (2019). Antioxidant enzymes regulation in plants in reference to reactive oxygen species (ROS) and reactive nitrogen species (RNS). Plant Gene.

[45] David L., Harmon A.C., Chen S. (2019). Plant immune responses—From guard cells and local responses to systemic defense against bacterial pathogens. Plant Signal Behav..

[46] Vlot A.C., Sales J.H., Lenk M., Bauer K., Brambilla A., Sommer A., Chen Y., Wenig M., Nayem S. (2021). Systemic propagation of immunity in plants. New Phytol..

[47] Miedes E., Vanholme R., Boerjan W., Molina A. (2014). The role of the secondary cell wall in plant resistance to pathogens. Front. Plant Sci..

[48] Zhang R., Zheng F., Wei S., Zhang S., Li G., Cao P., Zhao S. (2019). Evolution of disease defense genes and their regulators in plants. Int. J. Mol. Sci..

[49] Mhamdi A., Van Breusegem F. (2018). Reactive oxygen species in plant development. Development.

[50] Kunkel B., Harper C. (2017). The roles of auxin during interactions between bacterial plant pathogens and their hosts. J. Exp. Bot..

[51] Djami-Tchatchou A.T., Harrison G.A., Harper C.P., Wang R., Prigge M.J., Estelle M., Kunkel B.N. (2020). Dual role of auxin in regulating plant defense and bacterial virulence gene expression during *Pseudomonas syringae* PtoDC3000 pathogenesis. Mol. Plant Microbe Interact..

[52] Zhang Q., Gong M., Xu X., Li H., Deng W. (2022). Roles of auxin in the growth, development, and stress tolerance of horticultural plants. Cells.

[53] Zou X., Long J., Zhao K., Peng A., Chen M., Long Q., He Y., Chen S. (2019). Overexpressing *GH3.1* and *GH3.1L* reduces susceptibility to *Xanthomonas citri* subsp. *citri* by repressing auxin signaling in citrus (*Citrus sinensis* Osbeck). PLoS ONE.

[54] Kazan K., Lyons R. (2014). Intervention of phytohormone pathways by pathogen effectors. Plant Cell.

[55] Robert-Seilaniantz A., Grant M., Jones J.D. (2011). Hormone crosstalk in plant disease and defense: More than just jasmonate-salicylate antagonism. Annu. Rev. Phytopathol..

[56] Iglesias M.J., Terrile M.C., Casalongué C.A. (2011). Auxin and salicylic acid signalings counteract the regulation of adaptive responses to stress. Plant Signal Behav..

[57] Shasmita, Mohapatra D., Mohapatra P.K., Naik S.K., Mukherjee A.K. (2019). Priming with salicylic acid induces defense against bacterial blight disease by modulating rice plant photosystem II and antioxidant enzymes activity. Physiol. Mol. Plant Pathol..

[58] Dignam B.E.A., Marshall S.D.G., Wall A.J., Mtandavari Y.F., Gerard E.M., Hicks E., Cameron C., Aalders L.T., Shi S., Bell N.L. (2022). Impacts of soil-borne disease on plant yield and farm profit in dairying soils. J. Sustain. Agric. Environ..

[59] Pagán I., García-Arenal F. (2018). Tolerance to plant pathogens: Theory and experimental evidence. Int. J. Mol. Sci..

[60] Georgieva M., Vassileva V. (2023). Stress management in plants: Examining provisional and unique dose-dependent responses. Int. J. Mol. Sci..

[61] Chaloner T.M., Gurr S.J., Bebber D.P. (2021). Plant pathogen infection risk tracks global crop yields under climate change. Nat. Clim. Chang..

